# Relativistic Prolapse-Free Gaussian Basis Sets of Double- and Triple‑ζ Quality for *d*‑Block Elements: (aug-)RPF-2Z and (aug-)RPF-3Z

**DOI:** 10.1021/acs.jctc.5c00669

**Published:** 2025-08-06

**Authors:** Julielson dos Santos Sousa, Anne Kéllen de Nazaré dos Reis Dias, Eriosvaldo Florentino Gusmão, Roberto Luiz Andrade Haiduke

**Affiliations:** Department of Chemistry and Molecular Physics, São Carlos Institute of Chemistry, 28133 University of São Paulo, São Carlos, São Paulo 13566-590, Brazil

## Abstract

This study introduces new series of relativistic prolapse-free Gaussian basis sets of double- and triple-ζ quality (RPF-2Z and RPF-3Z), specifically designed for all known *d*-block elements. The polynomial version of the Generator Coordinate Dirac–Fock (p-GCDF) method is employed, along with multireference configuration interaction calculations with single and double substitutions (MR-CISD), to obtain correlation/polarization functions. Augmented versions containing additional diffuse functions for all orbital symmetries (aug-RPF-2Z and aug-RPF-3Z) are also provided. The quality of the basis sets was adequately validated through relativistic coupled-cluster calculations performed for equilibrium bond lengths, harmonic vibrational frequencies, and dipole moments of selected diatomic systems. The comparative analysis reveals some computational efficiency advantages with respect to traditional sets of similar sizes, such as dyall.v2z and dyall.v3z.

## Graphical abstract

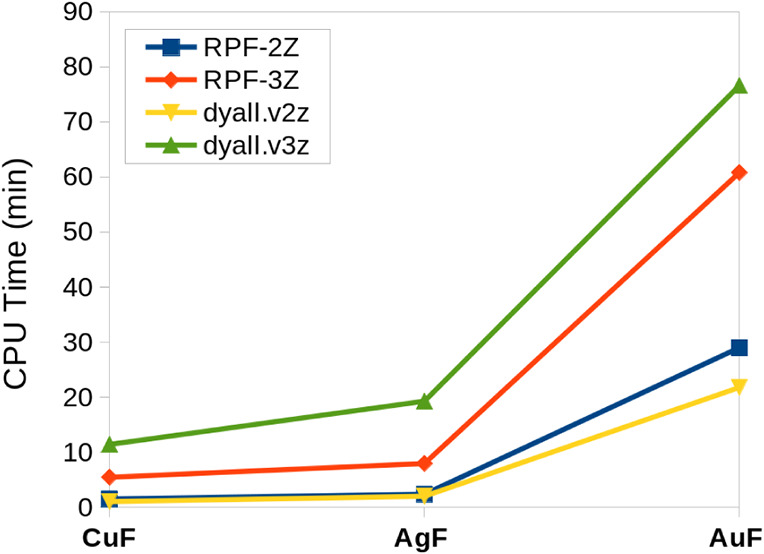

## 1 Introduction

The development of relativistic basis sets for four-component calculations, which are based on the Dirac equation,^1^ is a fundamental step toward achieving efficient and accurate electronic structure descriptions in heavy-element compounds. The formalism proposed by Dirac introduces the concept of four-component spinors, explicitly incorporating spin–orbit coupling along with other important relativistic corrections. As a result of the application of relativistic quantum mechanics, several interesting examples describing relativistic effects in chemistry are now well known.^2^

Actually, basis sets developed to be used in four-component calculations are more easily found in the literature, although variational prolapse^3^ can be evidenced in most of them. This basis set incompleteness issue arises from an inadequate representation of spinors near the nuclear region and can result in nonphysically low electronic energies,^3,4^ although it is different from the quite serious variational collapse issue that was resolved by kinetic balance conditions.^3^ Thus, the implementation of specialized strategies is required for the prolapse solution. Initially, very large Gaussian basis sets were developed to mitigate this problem,^5,6^ although their size eventually prevented the usage of such sets in the majority of practical applications. Recently, a polynomial version of the Generator Coordinate Dirac–Fock (p-GCDF) method^7^ was used to provide prolapse-free basis sets of primitive Gaussian functions with much smaller sizes,^8–10^ which should be augmented by correlation/polarization (C/P) functions for general applications in atomic and molecular systems. This work has already been initiated for the known *s*- and *p*-block elements of the periodic table (up to Oganesson).^11^

Here, proceeding with the development of small- and medium-size relativistic prolapse-free basis sets of Gaussian functions—that is, double-ζ (RPF-2Z) and triple-ζ (RPF-3Z) quality alternatives for electronic structure calculations in atomic and molecular systems—this research now focuses on *d*-block elements. Furthermore, augmented versions with extra diffuse functions, aug-RPF-2Z and aug-RPF-3Z, are also designed. Comparative calculations done for selected molecules support the expected accuracy level of these new sets. Additionally, these prolapse-free sets present a fundamental advantage that should improve the description of properties more closely related to inner electron distributions as well.

## 2 Methodology

The methodology in this paper follows the same approach of previous works.^11–14^ Thus, each Gaussian-type function exponent for a specific angular symmetry *w* (γ_*i*_^(*w*)^)) is achieved using a polynomial version of the Generator Coordinate Dirac–Fock method (p-GCDF) truncated at the third order, that is,

1 $$\Theta_{i}^{\left(w\right)}=\frac{\ln\gamma_{i}^{\left(w\right)}}{\alpha }=\Theta_{min}^{\left(w\right)}+\overset{3}{\underset{q=1}{\sum }}\Delta \Theta_{q}^{\left(w\right)}{\left(i-1\right)}^{q}$$

and

2 $$\gamma_{i}^{\left(w\right)}=\exp\left\{\alpha \left[\Theta_{min}^{\left(w\right)}+\overset{3}{\underset{q=1}{\sum }}\Delta \Theta_{q}^{\left(w\right)}{\left(i-1\right)}^{q}\right]\right\}$$

where *i* = 1, 2,···, *N*, being *N* the number of discretization points, α is a scaling parameter, assumed as 6.0,^15^ while $\Theta_{\min}^{\left(w\right)}$ and $\Delta \Theta_{q}^{\left(w\right)}$ are parameters corresponding, respectively, to the initial point of the mesh and the increment of order *q* used to achieve the discretization points. These parameters and the primitive Gaussian functions taken as initial sets for augmentation are those from the small- and medium-size relativistic prolapse-free (SRPF and MRPF, respectively) basis sets developed previously^8–10^ for the experimental electron configuration of each element. The additional *C*/*P* functions were incorporated to account for describing correlation and polarization effects of valence electrons, trying to maintain the same basis set size along each *d* sub-block and keeping consistency with the RPF-4Z sets as well.^13^ These can be functions of larger angular momentum (*l*) than the ones already considered in the SRPF and MRPF sets, whose exponents are obtained from functions of *l*–2 angular momentum, or can be more diffuse functions of a certain angular momentum already available in SRPF and MRPF sets, which are achieved by extrapolation from the parameters of the respective symmetry by using *i* ≤ 0 values in eq 2. This strategy aims to reduce the number of *small* functions generated by the restricted kinetic balance conditions adopted here (upward and downward).

All calculations were carried out using the DIRAC 23 ^16,17^ computational package with the Dirac-Coulomb (DC) Hamiltonian, the Gaussian nuclear model, and the standard light speed value of 137.0359992 atomic units (*au*). In order to simplify the treatment of the interelectronic integrals between the *small* component functions [(*SS*|*SS*)-type integrals], an approximation was adopted.^18^ The basis sets were used in their uncontracted form, as recommended for calculations employing restricted kinetic balance conditions. Thus, only the *large* component functions are discussed throughout this work.

First, in order to select the *C*/*P* functions, the multireference configuration interaction method with single and double excitations (MR-CISD) was adopted to treat the correlation energy of the valence space through the direct relativistic configuration interaction (DIRRCI) module within the DIRAC23 code.^16^ Thus, we defined a restricted active space (RAS), considering all possible excitation schemes along the valence region (RAS2) and up to double substitutions to virtual spinors with energies of up to 20.0 *au* (RAS3). In more detail, the active space includes all (*n* – 1)*d* and *ns* valence electrons (with *n* = 4–7 for each period, respectively). Thus, the RAS1 space was not used here. Hence, the number of active electrons in each *d* sub-block increases monotonically from 3 to 12. Additionally, the Davidson correction was implemented whenever applicable.

The basis set quality assessment step was conducted using the relativistic Coupled Cluster method with single and double excitations, along with a perturbative treatment of triple excitations (DC–CCSD-T). This process involved the evaluation of fundamental molecular parameters, such as equilibrium bond lengths (*r*_*e*_), harmonic vibrational frequencies (ω_*e*_), and electric dipole moments (μ). Here, dipole moments are calculated at the experimental equilibrium geometries.^19,20^ Moreover, the *r*_*e*_ and ω_*e*_ determinations are performed by fitting a potential energy curve (PEC) represented by five energy values calculated around the minimum (separated by 0.01 Å) using a fourth-order polynomial.

Dipole moments are determined by adding the analytical result of this property, evaluated at the Dirac–Fock (DF) level, with the contribution of electronic correlation from the DC–CCSD-T calculations, which is achieved by using Response Theory with an appropriate perturbation and the two-point finite difference technique, namely,

3 $${\left(\frac{\partial E\left(\lambda \right)}{\partial \lambda }\right)}_{0}\approx -\left(\frac{E\left(+\lambda \right)-E\left(-\lambda \right)}{2\lambda }\right)$$

In this context, *E* represents the correlation energy, and λ is the intensity of the field, defined as 1 × 10^–6^ au. The active space for the calculations of molecular properties was defined to encompass all occupied and virtual spinors with energies in the range of −3 to 20 au (with a minimum gap of 1 au). This means that 12, 18, 18, 24, 34, and 40 active electrons are considered for CuH, CuF, AgH, AgF, AuH, and AuF molecules, respectively, independent of the basis set used.

## 3 Basis Set Augmentation with Correlation/Polarization Functions

### 3.1 RPF-2Z and RPF-3Z Sets

The RPF-2Z sets derived from SRPFs^8–10^ are discussed in Table 1. The *d*-block elements already contain occupied *p* and *d* spinors. Hence, as mentioned before, the possible augmentation by *C*/*P* functions of these symmetries is evaluated by considering more diffuse functions than those contained in the starting primitive sets (*i* ≤ 0 values in eq 2). First, two diffuse *p* functions (*i* = 0, −1) were included sequentially and, next, a more diffuse *d* (*i* = 0) function was selected to maintain the consistency with the RPF-4Z sets within these symmetries.^13^ Thus, an *f* function was also considered, where its exponent was retrieved from the *p* functions for 3*d* and 4*d* elements. Hence, the recommended *f* function was chosen as the one causing the largest decrease in the electronic energies from MR-CISD calculations. The *i* values of such functions are well-behaved along the periods, increasing from 1 to 2 at a certain intermediate element as the atomic number increases. Furthermore, a more diffuse *f* function is considered for the 5*d* and 6*d* block elements. The primitive set achieved for the electron configuration associated with the unusual experimental ground state of Palladium (^1^S_0_), which does not include as diffuse *s* functions as the sets obtained for the other elements in the 4*d* sub-block, requires some care. Therefore, an extra diffuse *s* function (*i* = 0) was also inserted into the RPF-2Z set of Pd for this reason (see Table 1).

**Table 1:** Values of *i* That Define the C/P Functions for the RPF-2Z Set of *d*-Block Elements According to Eqs. 1 and 2 and to Their P-GCDF parameters.^8–10^

	3*d*-block		4*d*-block^a^		5*d*-block	6*d*-block
*w*	Sc–Ti	V–Zn	Y–Nb	Mo–Cd	Lu–Hg	Lr-Cn
*p* ^b^	–1, 0	–1, 0	–1, 0	–1, 0	–1, 0	–1, 0
*d* ^c^	0	0	0	0	0	0
*f*	1^b^	2^b^	1^b^	2^b^	0^d^	0^d^

a The basis set of Pd was also complemented by one diffuse *s* function (*i* = 0) by using $\Theta_{min}^{\left(s\right)}$ and $\Delta \Theta_{q}^{\left(s\right)}$ parameters.

b The $\Theta_{min}^{\left(p\right)}$ and $\Delta \Theta_{q}^{\left(p\right)}$ parameters are used to obtain these function exponents.

c The $\Theta_{min}^{\left(d\right)}$ and $\Delta \Theta_{q}^{\left(d\right)}$ parameters are used to obtain these function exponents.

d The $\Theta_{min}^{\left(f\right)}$ and $\Delta \Theta_{q}^{\left(f\right)}$ parameters are used to obtain these function exponents.

The RPF-3Z basis sets derived from MRPFs^8–10^ are discussed in Table 2. Starting from the primitive Gaussian functions, a traditional triple-ζ augmentation for the lightest *d*-block elements usually requires the inclusion of two *f* plus one *g* functions. However, more diffuse functions in lower angular momentum symmetries may also be required due to their effects on total energies and to maintain consistency with the RPF-4Z sets,^13^ being added initially (2*p* and 1*d* functions are again recommended). Next, the effect of *f* function pairs on correlation energies is considered for selection and, finally, the *g* function is chosen. Thus, after a similar procedure used to achieve the RPF-2Z sets, the C/P sets for RPF-3Z are composed of [2*p*,1*d*,2*f*,1*g*] functions for 3*d* and 4*d* sub-blocks. However, only one *f* function is recommended for the heaviest *d*-elements that already contain occupied *f* spinors (5*d* and 6*d* sub-blocks) and is chosen as a more diffuse Gaussian function within this symmetry (*i* = 0), providing a *C*/*P* set of [2*p*,1*d*,1*f*,1*g*] functions.

**Table 2:** Values of *i* That Define the C/P Functions for the RPF-3Z Set of *d*-Block Elements According to Eqs. 1 and 2 and to Their P-GCDF parameters.^8–10^

	3*d*-block		4*d*-block^a^				5*d*-block		6*d*-block	
w	Sc–V	Cr–Zn	Y–Zr	Nb–Ru	Rh–Ag	Cd	Lu–Hf	Ta–Hg	Lr–Db	Sg–Cn
*p* ^b^	–1, 0	–1, 0	–1, 0	–1, 0	–1, 0	–1, 0	–1, 0	–1,0	–1, 0	–1,0
*d* ^c^	0	0	0	0	0	0	0	0	0	0
*f*	1, 3^b^	1, 3^b^	1, 2^b^	1, 2^b^	1, 2^b^	2, 3^b^	0^d^	0^d^	0^d^	0^d^
*g* ^c^	3	4	2	3	4	4	2	3	2	3

a The basis set of Pd was also complemented by one diffuse *s* function (*i* = 0) by using $\Theta_{min}^{\left(s\right)}$ and $\Delta \Theta_{q}^{\left(s\right)}$ parameters.

b The $\Theta_{min}^{\left(p\right)}$ and $\Delta \Theta_{q}^{\left(p\right)}$ parameters are used to obtain these function exponents.

c The $\Theta_{min}^{\left(d\right)}$ and $\Delta \Theta_{q}^{\left(d\right)}$ parameters are used to obtain these function exponents.

d The $\Theta_{min}^{\left(f\right)}$ and $\Delta \Theta_{q}^{\left(f\right)}$ parameters are used to obtain these function exponents.

In the case of *f*-type *C*/*P* functions for the 3*d* sub-block, the pair composed of *f*_1_ and *f*_3_ proved to be more efficient in describing correlation energies of valence electrons. Again, this pattern was maintained throughout the entire period. Subsequently, a study was conducted on the addition of one *g* function to the set, now already augmented by *f*_1_ and *f*_3_, resulting in the choice of *g*_3_ for the lightest 3*d* atoms (Sc–V), while a more compact function (*g*_4_) was required for the remaining ones (Cr–Zn).

As listed in Table 2, the pair given by *f*_1_ and *f*_2_ was chosen for almost all elements of the 4*d* sub-block except for Cd, which is better described by the *f*_2_ and *f*_3_ pair instead. Next, *g*_2_ was adopted for the lightest 4*d* atoms (Y and Zr), while *g*_3_ is the best choice for intermediate elements (Nb–Ru) and *g*_4_ was selected for the last ones (Rh–Cd). Similarly, in the 5*d* sub-block, the *g*_2_ function was more suitable for Lu and Hf, while *g*_3_ is recommended for the remaining elements, Ta—Hg. Furthermore, within the 6*d* sub-block, the best choice was *g*_2_ for Lr–Db and *g*_3_ for Sg–Cn. As noted before,^13^ the advantage of the polynomial expansions within the p-GCDF approach for the choice of *C*/*P* functions can be evidenced again along each *d* sub-block investigated here. Accordingly, the RPF-3Z set of Pd also incorporates one diffuse *s* function (*i* = 0) for the same reasons previously mentioned.

### 3.2 aug-RPF-2Z and aug-RPF-3Z Sets

Additional versions of the previous basis sets containing extra diffuse functions were also generated to be used in anions and other systems requiring such a type of augmentation. The exponents of these diffuse Gaussian functions are obtained by using *i* values one unit smaller than those previously considered in each symmetry already present in the original RPF-2Z and RPF-3Z sets (*s*, *p*, *d*, *f*, and *g*), providing, respectively, the aug-RPF-2Z and aug-RPF-3Z alternatives.

## 4 Molecular Calculations

In this section, we first address the results obtained from DC–CCSD-T molecular calculations performed using the RPF-2Z and RPF-3Z basis sets for equilibrium distances (*r*_*e*_), harmonic vibrational frequencies (ω_*e*_), and dipole moments (μ). These data are also compared with those obtained from the traditional cc-pVTZ set,^21,22^ as well as with those from dyall.v2z and dyall.v3z relativistic sets.^23–27^ Hence, the following molecules were considered: CuH, CuF, AgH, AgF, AuH, and AuF. The signed deviations of the calculated quantities with respect to experimental data^19,20,28^ are evaluated.

As one can see in Table 3, RPF-2Z presents molecular property results in fair agreement with the expectations for a set of double-ζ quality. In addition, RPF-3Z shows comparable performance with respect to dyall.v3z, with predictions that are also slightly more accurate than those from cc-pVTZ for CuH and CuF. The mean absolute deviations (MADs) obtained for *r*_*e*_ values decrease from 0.0124 to 0.0058 Å when moving from RPF-2Z to RPF-3Z. In addition, the MADs obtained for ω_*e*_ decrease from 30 to 24 cm^–1^ as one goes from RPF-2Z to RPF-3Z. These deviations are similar to those previously observed for the same two basis sets, considering a group of molecules containing only *s*- and *p*-block elements.^11^

**Table 3:** Results of DCCCSD-T Calculations for Equilibrium Bond Lengths (*r*_*e*_), Harmonic Vibrational Frequencies (ω_*e*_), and Molecular Dipole Moments (*μ*), Along with the Signed Deviations for Each Property (*y*) Obtained with Respect to Experimental Data [Dev­(y) = Calc­(y)—Exp­(y)] ^19,20,28^

		*r*_e_ (Å)		ω_e_ (cm^–1^)		μ (D)^a^		
Molecules	Sets	Calc.	Dev	Calc.	Dev	Calc.	Dev	Time (min)^b^
^63^CuH	RPF-2Z	1.451	–0.011	1985	44.0	2.82	–	0.67
	RPF-3Z	1.456	–0.006	1978	36.4	2.69	–	2.03
	cc-pVTZ	1.449	–0.014	1987	45.8	2.80	–	2.07
	dyall.v2z	1.464	0.001	1917	–24.2	2.77	–	0.40
	dyall.v3z	1.458	–0.005	1960	19.2	2.68	–	4.46
^63^Cu^19^F	RPF-2Z	1.755	0.010	599	–23.7	5.53	–0.24	1.51
	RPF-3Z	1.742	–0.002	623	–0.2	5.26	–0.51	5.45
	cc-pVTZ	1.733	–0.012	633	10.7	5.15	–0.62	5.54
	dyall.v2z	1.746	0.001	625	2.4	6.19	0.42	1.03
	dyall.v3z	1.739	–0.006	627	4.5	6.08	0.31	11.42
^107^AgH	RPF-2Z	1.599	–0.019	1810	49.8	2.93	–	1.08
	RPF-3Z	1.602	–0.016	1815	55.4	2.80	–	3.45
	dyall.v2z	1.623	0.005	1751	–9.1	2.80	–	0.90
	dyall.v3z	1.611	–0.007	1786	26.5	2.78	–	8.35
^107^Ag^19^F	RPF-2Z	2.005	0.022	492	–21.6	6.23	0.01	2.37
	RPF-3Z	1.986	0.003	515	1.4	6.00	–0.22	7.95
	dyall.v2z	2.000	0.017	509	–4.9	5.86	–0.36	2.00
	dyall.v3z	1.979	–0.004	518	4.9	5.80	–0.42	19.26
^197^AuH	RPF-2Z	1.522	–0.002	2341	35.7	1.26	–	14.27
	RPF-3Z	1.518	–0.006	2353	47.7	1.27	–	27.32
	dyall.v2z	1.529	0.005	2325	19.7	1.28	–	7.07
	dyall.v3z	1.521	–0.003	2324	19.4	1.26	–	34.64
^197^Au^19^F	RPF-2Z	1.930	0.011	557	–6.5	4.19	–	28.98
	RPF-3Z	1.918	–0.001	564	0.8	4.23	–	60.77
	dyall.v2z	1.935	0.017	546	–17.6	4.19	–	21.76
	dyall.v3z	1.917	–0.002	569	5.4	4.10	–	76.57

a CuH, AgH, AuH, and AuF are not included in the error determinations due to the lack of accurate experimental data.

b Total CPU time in DC–CCSD-T calculations with different basis sets performed in a Intel­(R) Xeon­(R) Gold 5318Y CPU @ 2.10 GHz processor.

Table 3 also displays the CPU times required for DC–CCSD-T calculations carried out on fixed molecular geometries. A comparative analysis reveals that RPF-2Z sets are a little more demanding than dyall.v2z for the investigated systems. In contrast, RPF-3Z basis sets consistently exhibit superior computational efficiency compared to dyall.v3z. In addition, cc-pVTZ provided a computational demand similar to that of the RPF-3Z set. Hence, these results suggest that prolapse-free basis sets can be computationally efficient, as well.

## 5 Conclusions

This work provides correlation/polarization (C/P) functions generated through the p-GCDF methodology to be used with SRPF and MRPF sets in order to address the properties of the valence electrons. Hence, this methodology yields relativistic prolapse-free Gaussian basis sets of double- and triple-ζ quality for *d*-block elements, referred here as RPF-2Z and RPF-3Z, respectively. Augmented versions containing extra diffuse functions for every symmetry (aug-RPF-2Z and aug-RPF-3Z) are also developed.

Molecular calculations confirm the accuracy and reliability of these sets, considering key properties such as equilibrium bond lengths, harmonic vibrational frequencies, and dipole moments. Finally, the computational efficiency of the new basis sets was evaluated by comparison with other alternatives such as dyall.v2z and dyall.v3z, indicating that RPF-2Z and RPF-3Z sets can also exhibit advantages in this aspect. Thus, these new basis sets are recommended to deal with general properties of atomic and molecular systems and, additionally, should provide a better description of the innermost spinors due to the absence of variational prolapse issues.

## Supplementary Material











## Notes

Five files containing energies obtained in atomic calculations, along with the RPF-2Z, RPF-3Z, aug-RPF-2Z, and aug-RPF-3Z sets are available (PDF)
Relativistic prolapse-free Gaussian basis sets of double-zeta quality: RPF-2Z (TXT)
Relativistic prolapse-free Gaussian basis sets of triple-zeta quality: RPF-3Z (TXT)
Augmented relativistic prolapse-free Gaussian basis sets of double-zeta quality: aug-RPF-2Z (TXT)
Augmented relativistic prolapse-free Gaussian basis sets of triple-zeta quality: aug-RPF-3Z (TXT)
