# Supplementary material for: Relativistic Prolapse-Free Gaussian Basis Sets of Double- and Triple‑ζ Quality for d‑Block Elements: (aug-)RPF-2Z and (aug-)RPF-3Z
Source: J Chem Theory Comput. 2025 Aug 6;21(16):7813–7. doi: 10.1021/acs.jctc.5c00669 (PMC12392452; doi:10.1021/acs.jctc.5c00669)
Supplement: Supplementary file 1 [file ct5c00669_si_001.pdf]

Relativistic Prolapse-Free Gaussian Basis Sets of Double- and Triple-ζ Quality for d-Block Elements: (aug-)RPF-2Z and (aug-)RPF-3Z

Julielson dos Santos Sousa, Anne Kéllen de Nazaré dos Reis Dias, Eriosvaldo Florentino Gusmão, and Roberto Luiz Andrade Haiduke

Department of Chemistry and Molecular Physics, São Carlos Institute of Chemistry, University of São Paulo, 13566-590, São Carlos, SP,Brazil.

E-mail: julielson@usp.br; Annekellenreis@gmail.com; eriosvaldo.gusmao@alumni.usp.br; haiduke@iqsc.usp.br

Table S1: Total and correlation energies (in Hartree) and the variations in these quantities due to basis set augmentation (Δ, in mHartree) of the RPF-2Z basis set (Davidson corrections are considered when available). The numbers between square brackets refer to the i values of the functions added.

21-Sc	Total Energy	Correlation Energy	Δ(Total Energy)	Δ(Corr. Energy)
Sc	-763.3885	-0.0616		
Sc+2p[-1,0]+d[0]	-763.4051	-0.0789	-16.7	-17.3
Sc+2p+1d+f[0]	-763.4068	-0.0806	-1.6	-1.6
**Sc+2p+1d+f[1]**	**-763.4089**	**-0.0827**	**-3.7**	**-3.7**
Sc+2p+1d+f[2]	-763.4075	-0.0813	-2.4	-2.4

22-Ti	Total Energy	Correlation Energy	Δ(Total Energy)	Δ(Corr. Energy)
Ti	-852.8708	-0.1020		
Ti+2p[-1,0]+d[0]	-852.8867	-0.1180	-15.9	-16.0
Ti+2p+1d+f[0]	-852.8888	-0.1201	-2.1	-2.1
**Ti+2p+1d+f[1]**	**-852.8933**	**-0.1245**	**-6.5**	**-6.5**
Ti+2p+1d+f[2]	-852.8926	-0.1238	-5.9	-5.9

23-V	Total Energy	Correlation Energy	Δ(Total Energy)	Δ(Corr. Energy)
V	-948.2956	-0.1219		
V+2p[-1,0]+d[0]	-948.3115	-0.1372	-15.9	-15.4
V+2p+1d+f[1]	-948.3209	-0.1466	-9.4	-9.4
**V+2p+1d+f[2]**	**-948.3231**	**-0.1488**	**-11.6**	**-11.6**
V+2p+1d+f[3]	-948.3178	-0.1435	-6.3	-6.3

24-Cr	Total Energy	Correlation Energy	Δ(Total Energy)	Δ(Corr. Energy)
Cr	-1049.8651	-0.1960		
Cr+2p[-1,0]+d[0]	-1049.8665	-0.1972	-1.4	-1.2
Cr+2p+1d+f[1]	-1049.8827	-0.2134	-16.2	-16.2
**Cr+2p+1d+f[2]**	**-1049.8907**	**-0.2215**	**-24.2**	**-24.2**
Cr+2p+1d+f[3]	-1049.8810	-0.2118	-14.6	-14.6

25-Mn	Total Energy	Correlation Energy	Δ(Total Energy)	Δ(Corr. Energy)
Mn	-1157.6131	-0.2696		
Mn+2p[-1,0]+d[0]	-1157.6320	-0.2870	-18.9	-17.4
Mn+2p+1d+f[1]	-1157.6477	-0.3027	-15.7	-15.7
**Mn+2p+1d+f[2]**	**-1157.6603**	**-0.3154**	**-28.3**	**-28.3**
Mn+2p+1d+f[3]	-1157.6525	-0.3075	-20.5	-20.5

26-Fe	Total Energy	Correlation Energy	Δ(Total Energy)	Δ(Corr. Energy)
Fe	-1271.6342	-0.2204		
Fe+2p[-1,0]+d[0]	-1271.6555	-0.2401	-21.3	-19.7

Fe+2p+1d+f[1]	-1271.6772	-0.2619	-21.8	-21.8
**Fe+2p+1d+f[2]**	**-1271.6955**	**-0.2802**	**-40.0**	**-40.0**
Fe+2p+1d+f[3]	-1271.6864	-0.2711	-31.0	-31.0

27-Co	Total Energy	Correlation Energy	Δ(Total Energy)	Δ(Corr. Energy)
Co	-1392.2318	-0.1994		
Co+2p[-1,0]+d[0]	-1392.2548	-0.2225	-23.0	-23.1
Co+2p+1d+f[1]	-1392.2825	-0.2502	-27.7	-27.7
**Co+2p+1d+f[2]**	**-1392.3076**	**-0.2753**	**-52.8**	**-52.8**
Co+2p+1d+f[3]	-1392.2981	-0.2657	-43.3	-43.3

28-Ni	Total Energy	Correlation Energy	Δ(Total Energy)	Δ(Corr. Energy)
Ni	-1519.5235	-0.1976		
Ni+2p[-1,0]+d[0]	-1519.5478	-0.2218	-24.4	-24.2
Ni+2p+1d+f[1]	-1519.5816	-0.2555	-33.7	-33.7
**Ni+2p+1d+f[2]**	**-1519.6151**	**-0.2890**	**-67.2**	**-67.2**
Ni+2p+1d+f[3]	-1519.6056	-0.2796	-57.8	-57.8

29-Cu	Total Energy	Correlation Energy	Davidson Corr.	Δ(Total Energy)	Δ(Corr.+Davidson)
Cu	-1653.6688	-0.2060	-0.0073		
Cu+2p[-1,0]+d[0]	-1653.7001	-0.2298	-0.0148	-31.3	-31.2
Cu+2p+1d+f[1]	-1653.7413	-0.2680	-0.0177	-41.1	-41.1
**Cu+2p+1d+f[2]**	**-1653.7871**	**-0.3122**	**-0.0193**	**-87.0**	**-87.0**
Cu+2p+1d+f[3]	-1653.7793	-0.3056	-0.0182	-79.2	-79.2

30-Zn	Total Energy	Correlation Energy	Davidson Corr.	Δ(Total Energy)	Δ(Corr.+Davidson)
Zn	-1794.8488	-0.2283	-0.0080		
Zn+2p[-1,0]+d[0]	-1794.8834	-0.2543	-0.0163	-34.6	-34.2
Zn+2p+1d+f[1]	-1794.9338	-0.3009	-0.0200	-50.4	-50.4
**Zn+2p+1d+f[2]**	**-1794.9895**	**-0.3549**	**-0.0218**	**-106.1**	**-106.1**
Zn+2p+1d+f[3]	-1794.9802	-0.3470	-0.0202	-96.7	-96.7

39-Y	Total Energy	Correlation Energy	Δ(Total Energy)	Δ(Corr. Energy)
Y	-3383.8141	-0.0338		
Y+2p[-1,0]+d[0]	-3383.8313	-0.0509	-17.3	-17.2
Y+2p+1d+f[0]	-3383.8344	-0.0540	-3.0	-3.0
**Y+2p+1d+f[1]**	**-3383.8369**	**-0.0564**	**-5.5**	**-5.5**
Y+2p+1d+f[2]	-3383.8339	-0.0535	-2.6	-2.6

40-Zr	Total Energy	Correlation Energy	Δ(Total Energy)	Δ(Corr. Energy)
Zr	-3597.1711	-0.0578		
Zr+2p[-1,0]+d[0]	-3597.1901	-0.0778	-19.0	-20.0
Zr+2p+1d+f[0]	-3597.1947	-0.0824	-4.6	-4.6

**Zr+2p+1d+f[1]**	**-3597.2020**	**-0.0898**	**-11.9**	**-11.9**
Zr+2p+1d+f[2]	-3597.1977	-0.0855	-7.7	-7.7
**41-Nb**	**Total Energy**	**Correlation Energy**	**Δ(Total Energy)**	**Δ(Corr. Energy)**
Nb	-3818.3233	-0.1041		
Nb+2p[-1,0]+d[0]	-3818.3255	-0.1061	-2.2	-2.0
Nb+2p+1d+f[0]	-3818.3322	-0.1128	-6.7	-6.7
**Nb+2p+1d+f[1]**	**-3818.3487**	**-0.1293**	**-23.3**	**-23.3**
Nb+2p+1d+f[2]	-3818.3467	-0.1273	-21.3	-21.3
**42-Mo**	**Total Energy**	**Correlation Energy**	**Δ(Total Energy)**	**Δ(Corr. Energy)**
Mo	-4047.4021	-0.1413		
Mo+2p[-1,0]+d[0]	-4047.4042	-0.1432	-2.1	-1.8
Mo+2p+1d+f[1]	-4047.4350	-0.1740	-30.8	-30.8
**Mo+2p+1d+f[2]**	**-4047.4396**	**-0.1786**	**-35.4**	**-35.4**
Mo+2p+1d+f[3]	-4047.4123	-0.1513	-8.2	-8.2
**43-Tc**	**Total Energy**	**Correlation Energy**	**Δ(Total Energy)**	**Δ(Corr. Energy)**
Tc	-4284.3898	-0.1355		
Tc+2p[-1,0]+d[0]	-4284.4118	-0.1558	-22.0	-20.4
Tc+2p+1d+f[1]	-4284.4438	-0.1878	-32.0	-32.0
**Tc+2p+1d+f[2]**	**-4284.4560**	**-0.2000**	**-44.2**	**-44.2**
Tc+2p+1d+f[3]	-4284.4245	-0.1686	-12.7	-12.7
**44-Ru**	**Total Energy**	**Correlation Energy**	**Δ(Total Energy)**	**Δ(Corr. Energy)**
Ru	-4529.4934	-0.1006		
Ru+2p[-1,0]+d[0]	-4529.5000	-0.1068	-6.6	-6.2
Ru+2p+1d+f[1]	-4529.5536	-0.1604	-53.6	-53.6
**Ru+2p+1d+f[2]**	**-4529.5736**	**-0.1805**	**-73.7**	**-73.7**
Ru+2p+1d+f[3]	-4529.5216	-0.1285	-21.7	-21.7
**45-Rh**	**Total Energy**	**Correlation Energy**	**Δ(Total Energy)**	**Δ(Corr. Energy)**
Rh	-4782.8582	-0.1075		
Rh+2p[-1,0]+d[0]	-4782.8657	-0.1146	-7.5	-7.1
Rh+2p+1d+f[1]	-4782.9301	-0.1790	-64.4	-64.4
**Rh+2p+1d+f[2]**	**-4782.9624**	**-0.2113**	**-96.7**	**-96.7**
Rh+2p+1d+f[3]	-4782.8974	-0.1463	-31.7	-31.7
**46-Pd**	**Total Energy**	**Correlation Energy**	**Δ(Total Energy)**	**Δ(Corr. Energy)**
Pd	-5044.6111	-0.1221		
Pd+2p[-1,0]+d[0]	-5044.6141	-0.1246	-3.0	-2.5
Pd+2p+1d+f[1]	-5044.6881	-0.1985	-73.9	-73.9
**Pd+2p+1d+f[2]**	**-5044.7431**	**-0.2536**	**-129.0**	**-129.0**

Pd+2p+1d+f[3]	-5044.6605	-0.1710		-46.4	-46.4
Pd+2p+1d+1f+s[0]	-5044.7438	-0.2543		-0.7	-0.7
**47-Ag**	**Total Energy**	**Correlation Energy**	**Davidson Corr.**	**Δ(Total Energy)**	**Δ(Corr.+Davidson)**
Ag	-5314.8512	-0.1511	-0.0087		
Ag+2p[-1,0]+d[0]	-5314.8620	-0.1591	-0.0112	-10.8	-10.5
Ag+2p+1d+f[1]	-5314.9570	-0.2448	-0.0204	-94.9	-94.9
**Ag+2p+1d+f[2]**	**-5315.0217**	**-0.3088**	**-0.0211**	**-159.7**	**-159.7**
Ag+2p+1d+f[3]	-5314.9213	-0.2157	-0.0138	-59.3	-59.3
**48-Cd**	**Total Energy**	**Correlation Energy**	**Davidson Corr.**	**Δ(Total Energy)**	**Δ(Corr.+Davidson)**
Cd	-5593.5499	-0.1147	-0.0030		
Cd+2p[-1,0]+d[0]	-5593.5840	-0.1421	-0.0093	-34.1	-33.7
Cd+2p+1d+f[1]	-5593.6785	-0.2296	-0.0164	-94.5	-94.5
**Cd+2p+1d+f[2]**	**-5593.7656**	**-0.3126**	**-0.0205**	**-181.6**	**-181.6**
Cd+2p+1d+f[3]	-5593.6587	-0.2131	-0.0130	-74.7	-74.7
**71-Lu**	**Total Energy**	**Correlation Energy**		**Δ(Total Energy)**	**Δ(Corr. Energy)**
Lu	-14573.7401	-0.0310			
Lu+2p[-1,0]+d[0]	-14573.7619	-0.0509		-21.8	-19.9
Lu+2p+1d+f[0]	-14573.7649	-0.0535		-3.0	-2.6
**72-Hf**	**Total Energy**	**Correlation Energy**		**Δ(Total Energy)**	**Δ(Corr. Energy)**
Hf	-15090.1382	-0.0548			
Hf+2p[-1,0]+d[0]	-15090.1630	-0.0789		-24.8	-24.1
Hf+2p+1d+f[0]	-15090.1687	-0.0840		-5.7	-5.1
**73-Ta**	**Total Energy**	**Correlation Energy**		**Δ(Total Energy)**	**Δ(Corr. Energy)**
Ta	-15618.1479	-0.0969			
Ta+2p[-1,0]+d[0]	-15618.1727	-0.1210		-24.8	-24.1
Ta+2p+1d+f[0]	-15618.1804	-0.1283		-7.7	-7.3
**74-W**	**Total Energy**	**Correlation Energy**		**Δ(Total Energy)**	**Δ(Corr. Energy)**
W	-16157.8881	-0.1274			
W+2p[-1,0]+d[0]	-16157.9127	-0.1511		-24.6	-23.7
W+2p+1d+f[0]	-16157.9231	-0.1612		-10.5	-10.1
**75-Re**	**Total Energy**	**Correlation Energy**		**Δ(Total Energy)**	**Δ(Corr. Energy)**
Re	-16709.5246	-0.2022			
Re+2p[-1,0]+d[0]	-16709.5507	-0.2271		-26.1	-24.9

Re+2p+1d+f[0]	-16709.5590	-0.2354		-8.3	-8.3
**76-Os**	**Total Energy**	**Correlation Energy**		**Δ(Total Energy)**	**Δ(Corr. Energy)**
Os	-17273.1235	-0.1873			
Os+2p[-1,0]+d[0]	-17273.1506	-0.2115		-27.0	-24.2
Os+2p+1d+f[0]	-17273.1588	-0.2197		-8.3	-8.3
**77-Ir**	**Total Energy**	**Correlation Energy**		**Δ(Total Energy)**	**Δ(Corr. Energy)**
Ir	-17848.9742	-0.1762			
Ir+2p[-1,0]+d[0]	-17849.0010	-0.2016		-26.8	-25.4
Ir+2p+1d+f[0]	-17849.0293	-0.2290		-28.3	-27.5
**78-Pt**	**Total Energy**	**Correlation Energy**		**Δ(Total Energy)**	**Δ(Corr. Energy)**
Pt	-18437.2260	-0.2100			
Pt+2p[-1,0]+d[0]	-18437.2514	-0.2342		-25.4	-24.2
Pt+2p+1d+f[0]	-18437.2794	-0.2619		-28.0	-27.7
**79-Au**	**Total Energy**	**Correlation Energy**	**Davidson Corr.**	**Δ(Total Energy)**	**Δ(Corr.+Davidson)**
Au	-19038.1187	-0.2770	-0.0161		
Au+2p[-1,0]+d[0]	-19038.1365	-0.2889	-0.0200	-17.8	-15.8
Au+2p+1d+f[0]	-19038.1737	-0.3195	-0.0264	-37.2	-37.0
**80-Hg**	**Total Energy**	**Correlation Energy**	**Davidson Corr.**	**Δ(Total Energy)**	**Δ(Corr.+Davidson)**
Hg	-19651.6455	-0.3914	-0.0532		
Hg+2p[-1,0]+d[0]	-19651.6762	-0.3253	-0.0270	-30.7	92.2
Hg+2p+1d+f[0]	-19651.7046	-0.3484	-0.0323	-28.4	-28.3
**103 – Lr**	**Total Energy**	**Correlation Energy**		**Δ(Total Energy)**	**Δ(Corr. Energy)**
Lr	-37720.2414	-0.0264			
Lr+2p[-1,0]+d[0]	-37720.2643	-0.0436		-22.9	-17.2
Lr+2p+1d+f[0]	-37720.2693	-0.0471		-5.0	-3.6
**104 - Rf**	**Total Energy**	**Correlation Energy**		**Δ(Total Energy)**	**Δ(Corr. Energy)**
Rf	-38707.2177	-0.0453			
Rf+2p[-1,0]+d[0]	-38707.2434	-0.0673		-25.7	-21.9
Rf+2p+1d+f[0]	-38707.2519	-0.0744		-8.6	-7.1
**105 - Db**	**Total Energy**	**Correlation Energy**		**Δ(Total Energy)**	**Δ(Corr. Energy)**

Db	-39713.7606	-0.0778			
Db+2p[-1,0]+d[0]	-39713.7907	-0.1023		-30.1	-24.5
Db+2p+1d+f[0]	-39713.8041	-0.1138		-13.4	-11.6
**106 - Sg**	**Total Energy**	**Correlation Energy**		**Δ(Total Energy)**	**Δ(Corr. Energy)**
Sg	-40740.5342	-0.1035			
Sg+2p[-1,0]+d[0]	-40740.5640	-0.1287		-29.8	-25.2
Sg+2p+1d+f[0]	-40740.5819	-0.1448		-17.9	-16.1
**107 - Bh**	**Total Energy**	**Correlation Energy**		**Δ(Total Energy)**	**Δ(Corr. Energy)**
Bh	-41788.2510	-0.1455			
Bh+2p[-1,0]+d[0]	-41788.2793	-0.1684		-28.3	-22.9
Bh+2p+1d+f[0]	-41788.3061	-0.1920		-26.7	-23.5
**108 - Hs**	**Total Energy**	**Correlation Energy**		**Δ(Total Energy)**	**Δ(Corr. Energy)**
Hs	-42856.6914	-0.1478			
Hs+2p[-1,0]+d[0]	-42856.7203	-0.1711		-28.8	-23.3
Hs+2p+1d+f[0]	-42856.7506	-0.1997		-30.3	-28.6
**109 - Mt**	**Total Energy**	**Correlation Energy**		**Δ(Total Energy)**	**Δ(Corr. Energy)**
Mt	-43947.1887	-0.1461			
Mt+2p[-1,0]+d[0]	-43947.2182	-0.1694		-29.4	-23.3
Mt+2p+1d+f[0]	-43947.2730	-0.2170		-54.9	-47.7
**110 - Ds**	**Total Energy**	**Correlation Energy**		**Δ(Total Energy)**	**Δ(Corr. Energy)**
Ds	-45057.7820	-0.1796			
Ds+2p[-1,0]+d[0]	-45057.8097	-0.2010		-27.8	-21.4
Ds+2p+1d+f[0]	-45057.8646	-0.2519		-54.9	-51.0
**111 - Rg**	**Total Energy**	**Correlation Energy**	**Davidson Corr.**	**Δ(Total Energy)**	**Δ(Corr.+Davidson)**
Rg	-46193.2465	-0.2165	-0.0108		
Rg+2p[-1,0]+d[0]	-46193.2795	-0.2366	-0.0169	-33.0	-26.2
Rg+2p+1d+f[0]	-46193.3420	-0.2885	-0.0254	-62.5	-60.4
**112 - Cn**	**Total Energy**	**Correlation Energy**	**Davidson Corr.**	**Δ(Total Energy)**	**Δ(Corr.+Davidson)**
Cn	-47352.3563	-0.2612	-0.0139		
Cn+2p[-1,0]+d[0]	-47352.3867	-0.2792	-0.0198	-30.4	-23.8
Cn+2p+1d+f[0]	-47352.4479	-0.3300	-0.0289	-61.2	-60.0

Table S2: Total and correlation energies (in Hartree) and the variations in these quantities due to basis set augmentation (Δ, in mHartree) of the RPF-3Z basis set (Davidson corrections are considered when available). The numbers between square brackets refer to the i values of the functions added.

21-Sc	Total Energy	Correlation Energy	Δ(Total Energy)	Δ(Corr. Energy)
Sc	-763.3921	-0.0637		
Sc+2p[-1,0]+d[0]	-763.4069	-0.0790	-14.8	-15.3
Sc+2p+1d+f[0]	-763.4083	-0.0804	-1.4	-1.4
Sc+2p+1d+f[1]	-763.4106	-0.0827	-3.7	-3.7
Sc+2p+1d+f[2]	-763.4097	-0.0818	-2.7	-2.7
Sc+2p+1d+f[3]	-763.4079	-0.0801	-1.0	-1.0
Sc+2p+1d+2f[0,1]	-763.4108	-0.0830	-3.9	-3.9
Sc+2p+1d+2f[0,2]	-763.4109	-0.0830	-4.0	-4.0
Sc+2p+1d+2f[0,3]	-763.4094	-0.0815	-2.4	-2.4
Sc+2p+1d+2f[1,2]	-763.4114	-0.0835	-4.5	-4.5
**Sc+2p+1d+2f[1,3]**	**-763.4114**	**-0.0836**	**-4.5**	**-4.5**
Sc+2p+1d+2f[2,3]	-763.4098	-0.0819	-2.8	-2.8
Sc+2p+1d+2f+g[2]	-763.4118	-0.0839	-0.3	-0.3
**Sc+2p+1d+2f+g[3]**	**-763.4118**	**-0.0840**	**-0.4**	**-0.4**
Sc+2p+1d+2f+g[4]	-763.4117	-0.0838	-0.2	-0.2

22-Ti	Total Energy	Correlation Energy	Δ(Total Energy)	Δ(Corr. Energy)
Ti	-852.8746	-0.1036		
Ti+2p[-1,0]+d[0]	-852.8888	-0.1182	-14.2	-14.5
Ti+2p+1d+f[0]	-852.8905	-0.1199	-1.8	-1.8
Ti+2p+1d+f[1]	-852.8949	-0.1243	-6.1	-6.1
Ti+2p+1d+f[2]	-852.8951	-0.1245	-6.4	-6.4
Ti+2p+1d+f[3]	-852.8919	-0.1213	-3.1	-3.1
Ti+2p+1d+2f[0,1]	-852.8952	-0.1246	-6.4	-6.4
Ti+2p+1d+2f[0,2]	-852.8967	-0.1261	-7.9	-7.9
Ti+2p+1d+2f[0,3]	-852.8937	-0.1231	-5.0	-5.0
Ti+2p+1d+2f[1,2]	-852.8977	-0.1271	-8.9	-8.9
**Ti+2p+1d+2f[1,3]**	**-852.8979**	**-0.1273**	**-9.1**	**-9.1**
Ti+2p+1d+2f[2,3]	-852.8959	-0.1253	-7.1	-7.1
Ti+2p+1d+2f+g[2]	-852.8986	-0.1280	-0.7	-0.7
**Ti+2p+1d+2f+g[3]**	**-852.8990**	**-0.1284**	**-1.1**	**-1.1**
Ti+2p+1d+2f+g[4]	-852.8988	-0.1282	-0.9	-0.9

23-V	Total Energy	Correlation Energy	Δ(Total Energy)	Δ(Corr. Energy)
V	-948.2991	-0.1229		
V+2p[-1,0]+d[0]	-948.3138	-0.1374	-14.7	-14.6
V+2p+1d+f[0]	-948.3160	-0.1396	-2.2	-2.2
V+2p+1d+f[1]	-948.3224	-0.1460	-8.5	-8.5
V+2p+1d+f[2]	-948.3257	-0.1493	-11.8	-11.8
V+2p+1d+f[3]	-948.3217	-0.1453	-7.8	-7.8
V+2p+1d+2f[0,1]	-948.3227	-0.1463	-8.9	-8.9
V+2p+1d+2f[0,2]	-948.3276	-0.1512	-13.7	-13.7
V+2p+1d+2f[0,3]	-948.3240	-0.1476	-10.2	-10.2
V+2p+1d+2f[1,2]	-948.3288	-0.1524	-14.9	-14.9
**V+2p+1d+2f[1,3]**	**-948.3303**	**-0.1539**	**-16.5**	**-16.5**

V+2p+1d+2f[2,3]	-948.3284	-0.1520	-14.6	-14.6
V+2p+1d+2f+g[2]	-948.3312	-0.1548	-0.9	-0.9
**V+2p+1d+2f+g[3]**	**-948.3319**	**-0.1555**	**-1.5**	**-1.5**
V+2p+1d+2f+g[4]	-948.3319	-0.1555	-1.5	-1.5
**24-Cr**	**Total Energy**	**Correlation Energy**	**Δ(Total Energy)**	**Δ(Corr. Energy)**
Cr	-1049.8683	-0.1965		
Cr+2p[-1,0]+d[0]	-1049.8693	-0.1974	-1.0	-0.9
Cr+2p+1d+f[1]	-1049.8837	-0.2119	-14.5	-14.5
Cr+2p+1d+f[2]	-1049.8934	-0.2215	-24.1	-24.1
Cr+2p+1d+f[3]	-1049.8869	-0.2150	-17.6	-17.6
Cr+2p+1d+f[4]	-1049.8749	-0.2030	-5.6	-5.6
Cr+2p+1d+2f[1,2]	-1049.8987	-0.2268	-29.4	-29.4
**Cr+2p+1d+2f[1,3]**	**-1049.9033**	**-0.2314**	**-34.0**	**-34.0**
Cr+2p+1d+2f[1,4]	-1049.8907	-0.2188	-21.4	-21.4
Cr+2p+1d+2f[2,3]	-1049.9006	-0.2288	-31.3	-31.3
Cr+2p+1d+2f[2,4]	-1049.9010	-0.2292	-31.8	-31.8
Cr+2p+1d+2f[3,4]	-1049.8878	-0.2159	-18.5	-18.5
Cr+2p+1d+2f+g[3]	-1049.9048	-0.2329	-1.5	-1.5
**Cr+2p+1d+2f+g[4]**	**-1049.9058**	**-0.2340**	**-2.5**	**-2.5**
Cr+2p+1d+2f+g[5]	-1049.9052	-0.2334	-1.9	-1.9
**25-Mn**	**Total Energy**	**Correlation Energy**	**Δ(Total Energy)**	**Δ(Corr. Energy)**
Mn	-1157.6176	-0.2705		
Mn+2p[-1,0]+d[0]	-1157.6348	-0.2871	-17.2	-16.6
Mn+2p+1d+f[1]	-1157.6486	-0.3009	-13.8	-13.8
Mn+2p+1d+f[2]	-1157.6619	-0.3142	-27.2	-27.2
Mn+2p+1d+f[3]	-1157.6589	-0.3112	-24.1	-24.1
Mn+2p+1d+f[4]	-1157.6436	-0.2959	-8.8	-8.8
Mn+2p+1d+2f[1,2]	-1157.6663	-0.3186	-31.5	-31.5
**Mn+2p+1d+2f[1,3]**	**-1157.6739**	**-0.3262**	**-39.1**	**-39.1**
Mn+2p+1d+2f[1,4]	-1157.6589	-0.3112	-24.1	-24.1
Mn+2p+1d+2f[2,3]	-1157.6726	-0.3249	-37.8	-37.8
Mn+2p+1d+2f[2,4]	-1157.6731	-0.3254	-38.3	-38.3
Mn+2p+1d+2f[3,4]	-1157.6577	-0.3100	-22.9	-22.9
Mn+2p+1d+2f+g[3]	-1157.6764	-0.3287	-2.5	-2.5
**Mn+2p+1d+2f+g[4]**	**-1157.6771**	**-0.3294**	**-3.1**	**-3.1**
Mn+2p+1d+2f+g[5]	-1157.6762	-0.3285	-2.2	-2.2
**26-Fe**	**Total Energy**	**Correlation Energy**	**Δ(Total Energy)**	**Δ(Corr. Energy)**
Fe	-1271.6392	-0.2215		
Fe+2p[-1,0]+d[0]	-1271.6585	-0.2401	-19.2	-18.6
Fe+2p+1d+f[1]	-1271.6775	-0.2591	-19.1	-19.1
Fe+2p+1d+f[2]	-1271.6964	-0.2780	-37.9	-37.9
Fe+2p+1d+f[3]	-1271.6942	-0.2758	-35.7	-35.7
Fe+2p+1d+f[4]	-1271.6728	-0.2544	-14.4	-14.4
Fe+2p+1d+2f[1,2]	-1271.7026	-0.2842	-44.1	-44.1
**Fe+2p+1d+2f[1,3]**	**-1271.7149**	**-0.2965**	**-56.5**	**-56.5**
Fe+2p+1d+2f[1,4]	-1271.6939	-0.2755	-35.4	-35.4
Fe+2p+1d+2f[2,3]	-1271.7136	-0.2953	-55.2	-55.2

Fe+2p+1d+2f[2,4]	-1271.7142	-0.2958	-55.7	-55.7
Fe+2p+1d+2f[3,4]	-1271.6924	-0.2740	-33.9	-33.9
Fe+2p+1d+2f+g[3]	-1271.7198	-0.3014	-4.9	-4.9
**Fe+2p+1d+2f+g[4]**	**-1271.7218**	**-0.3034**	**-6.9**	**-6.9**
Fe+2p+1d+2f+g[5]	-1271.7199	-0.3015	-5.0	-5.0

27-Co	Total Energy	Correlation Energy	Δ(Total Energy)	Δ(Corr. Energy)
Co	-1392.2368	-0.2008		
Co+2p[-1,0]+d[0]	-1392.2577	-0.2219	-21.0	-21.0
Co+2p+1d+f[1]	-1392.2821	-0.2462	-24.4	-24.4
Co+2p+1d+f[2]	-1392.3075	-0.2717	-49.8	-49.8
Co+2p+1d+f[3]	-1392.3067	-0.2708	-48.9	-48.9
Co+2p+1d+f[4]	-1392.2785	-0.2427	-20.8	-20.8
Co+2p+1d+2f[1,2]	-1392.3156	-0.2797	-57.9	-57.9
**Co+2p+1d+2f[1,3]**	**-1392.3333**	**-0.2974**	**-75.6**	**-75.6**
Co+2p+1d+2f[1,4]	-1392.3056	-0.2697	-47.9	-47.9
Co+2p+1d+2f[2,3]	-1392.3323	-0.2964	-74.5	-74.5
Co+2p+1d+2f[2,4]	-1392.3330	-0.2972	-75.3	-75.3
Co+2p+1d+2f[3,4]	-1392.3041	-0.2682	-46.4	-46.4
Co+2p+1d+2f+g[2]	-1392.3358	-0.2999	-2.5	-2.5
Co+2p+1d+2f+g[3]	-1392.3397	-0.3039	-6.4	-6.4
**Co+2p+1d+2f+g[4]**	**-1392.3427**	**-0.3069**	**-9.4**	**-9.4**

28-Ni	Total Energy	Correlation Energy	Δ(Total Energy)	Δ(Corr. Energy)
Ni	-1519.5287	-0.1988		
Ni+2p[-1,0]+d[0]	-1519.5508	-0.2208	-22.1	-22.0
Ni+2p+1d+f[1]	-1519.5805	-0.2505	-29.7	-29.7
Ni+2p+1d+f[2]	-1519.6140	-0.2839	-63.1	-63.1
Ni+2p+1d+f[3]	-1519.6153	-0.2853	-64.5	-64.5
Ni+2p+1d+f[4]	-1519.5794	-0.2494	-28.6	-28.6
Ni+2p+1d+2f[1,2]	-1519.6238	-0.2938	-73.0	-73.0
**Ni+2p+1d+2f[1,3]**	**-1519.6480**	**-0.3180**	**-97.2**	**-97.2**
Ni+2p+1d+2f[1,4]	-1519.6125	-0.2825	-61.7	-61.7
Ni+2p+1d+2f[2,3]	-1519.6476	-0.3175	-96.7	-96.7
Ni+2p+1d+2f[2,4]	-1519.6194	-0.2893	-68.6	-68.6
Ni+2p+1d+2f[3,4]	-1519.6119	-0.2819	-61.1	-61.1
Ni+2p+1d+2f+g[2]	-1519.6508	-0.3208	-2.8	-2.8
Ni+2p+1d+2f+g[3]	-1519.6558	-0.3257	-7.7	-7.7
**Ni+2p+1d+2f+g[4]**	**-1519.6600**	**-0.3300**	**-12.0**	**-12.0**

29-Cu	Total Energy	Correlation Energy	Davidson Corr.	Δ(Total Energy)	Δ(Corr.+Davidson)
Cu	-1653.7235	-0.2530	-0.0119		
Cu+2p[-1,0]+d[0]	-1653.7310	-0.2584	-0.0138	-7.5	-7.3
Cu+2p+1d+f[1]	-1653.7732	-0.2967	-0.0177	-42.3	-42.3
Cu+2p+1d+f[2]	-1653.8197	-0.3421	-0.0189	-88.8	-88.8
Cu+2p+1d+f[3]	-1653.8217	-0.3454	-0.0176	-90.7	-90.7
Cu+2p+1d+2f[1,2]	-1653.8347	-0.3551	-0.0209	-103.8	-103.8
**Cu+2p+1d+2f[1,3]**	**-1653.8704**	**-0.3893**	**-0.0224**	**-139.5**	**-139.5**
Cu+2p+1d+2f[2,3]	-1653.8687	-0.3892	-0.0208	-137.8	-137.8

Cu+2p+1d+2f+g[2]	-1653.8729	-0.3915	-0.0227	-2.5	-2.5
Cu+2p+1d+2f+g[3]	-1653.8787	-0.3971	-0.0229	-8.3	-8.3
**Cu+2p+1d+2f+g[4]**	**-1653.8853**	**-0.4034**	**-0.0232**	**-14.8**	**-14.8**

**30-Zn**	**Total Energy**	**Correlation Energy**	**Davidson Corr.**	**Δ(Total Energy)**	**Δ(Corr.+Davidson)**
Zn	-1794.8673	-0.2408	-0.0088		
Zn+2p[-1,0]+d[0]	-1794.8987	-0.2640	-0.0167	-31.4	-31.2
Zn+2p+1d+f[1]	-1794.9428	-0.3047	-0.0202	-44.1	-44.1
Zn+2p+1d+f[2]	-1794.9973	-0.3572	-0.0221	-98.6	-98.6
Zn+2p+1d+f[3]	-1795.0051	-0.3660	-0.0212	-106.5	-106.5
Zn+2p+1d+2f[1,2]	-1795.0122	-0.3704	-0.0238	-113.5	-113.5
**Zn+2p+1d+2f[1,3]**	**-1795.0547**	**-0.4112**	**-0.0256**	**-156.1**	**-156.1**
Zn+2p+1d+2f[2,3]	-1795.0547	-0.4123	-0.0245	-156.0	-156.0
Zn+2p+1d+2f+g[2]	-1795.0583	-0.4145	-0.0259	-3.6	-3.6
Zn+2p+1d+2f+g[3]	-1795.0650	-0.4208	-0.0262	-10.2	-10.2
**Zn+2p+1d+2f+g[4]**	**-1795.0713**	**-0.4270**	**-0.0264**	**-16.6**	**-16.6**

**39-Y**	**Total Energy**	**Correlation Energy**		**Δ(Total Energy)**	**Δ(Corr. Energy)**
Y	-3383.8182	-0.0354			
Y+2p[-1,0]+d[0]	-3383.8338	-0.0510		-15.7	-15.6
Y+2p+1d+f[0]	-3383.8367	-0.0539		-2.8	-2.8
Y+2p+1d+f[1]	-3383.8395	-0.0567		-5.6	-5.6
Y+2p+1d+f[2]	-3383.8369	-0.0541		-3.1	-3.1
Y+2p+1d+f[3]	-3383.8346	-0.0518		-0.7	-0.7
Y+2p+1d+2f[0,1]	-3383.8401	-0.0573		-6.2	-6.2
Y+2p+1d+2f[0,2]	-3383.8395	-0.0567		-5.7	-5.7
Y+2p+1d+2f[0,3]	-3383.8374	-0.0546		-3.6	-3.6
**Y+2p+1d+2f[1,2]**	**-3383.8402**	**-0.0574**		**-6.3**	**-6.3**
Y+2p+1d+2f[1,3]	-3383.8401	-0.0573		-6.3	-6.3
Y+2p+1d+2f[2,3]	-3383.8370	-0.0542		-3.1	-3.1
Y+2p+1d+2f+g[1]	-3383.8404	-0.0576		-0.2	-0.2
**Y+2p+1d+2f+g[2]**	**-3383.8408**	**-0.0580**		**-0.6**	**-0.6**
Y+2p+1d+2f+g[3]	-3383.8405	-0.0577		-0.4	-0.4

**40-Zr**	**Total Energy**	**Correlation Energy**		**Δ(Total Energy)**	**Δ(Corr. Energy)**
Zr	-3597.1755	-0.0602			
Zr+2p[-1,0]+d[0]	-3597.1925	-0.0779		-17.0	-17.7
Zr+2p+1d+f[0]	-3597.1966	-0.0820		-4.1	-4.1
Zr+2p+1d+f[1]	-3597.2042	-0.0896		-11.7	-11.7
Zr+2p+1d+f[2]	-3597.2012	-0.0867		-8.7	-8.7
Zr+2p+1d+f[3]	-3597.1947	-0.0802		-2.2	-2.2
Zr+2p+1d+2f[0,1]	-3597.2047	-0.0902		-12.2	-12.2
Zr+2p+1d+2f[0,2]	-3597.2050	-0.0904		-12.5	-12.5
Zr+2p+1d+2f[0,3]	-3597.1989	-0.0844		-6.4	-6.4
**Zr+2p+1d+2f[1,2]**	**-3597.2072**	**-0.0927**		**-14.7**	**-14.7**
Zr+2p+1d+2f[1,3]	-3597.2066	-0.0921		-14.1	-14.1
Zr+2p+1d+2f[2,3]	-3597.2015	-0.0869		-9.0	-9.0
Zr+2p+1d+2f+g[1]	-3597.2076	-0.0931		-0.4	-0.4
**Zr+2p+1d+2f+g[2]**	**-3597.2088**	**-0.0942**		**-1.5**	**-1.5**

Zr+2p+1d+2f+g[3]	-3597.2087	-0.0941	-1.5	-1.5

41-Nb	Total Energy	Correlation Energy	Δ(Total Energy)	Δ(Corr. Energy)
Nb	-3818.3260	-0.1044		
Nb+2p[-1,0]+d[0]	-3818.3277	-0.1061	-1.7	-1.7
Nb+2p+1d+f[0]	-3818.3338	-0.1121	-6.1	-6.1
Nb+2p+1d+f[1]	-3818.3497	-0.1280	-21.9	-21.9
Nb+2p+1d+f[2]	-3818.3511	-0.1295	-23.4	-23.4
Nb+2p+1d+f[3]	-3818.3341	-0.1124	-6.4	-6.4
Nb+2p+1d+2f[0,1]	-3818.3503	-0.1287	-22.6	-22.6
Nb+2p+1d+2f[0,2]	-3818.3572	-0.1355	-29.4	-29.4
Nb+2p+1d+2f[0,3]	-3818.3407	-0.1191	-13.0	-13.0
**Nb+2p+1d+2f[1,2]**	**-3818.3612**	**-0.1396**	**-33.5**	**-33.5**
Nb+2p+1d+2f[1,3]	-3818.3586	-0.1370	-30.9	-30.9
Nb+2p+1d+2f[2,3]	-3818.3521	-0.1304	-24.3	-24.3
Nb+2p+1d+2f+g[2]	-3818.3623	-0.1407	-1.1	-1.1
**Nb+2p+1d+2f+g[3]**	**-3818.3637**	**-0.1421**	**-2.5**	**-2.5**
Nb+2p+1d+2f+g[4]	-3818.3630	-0.1414	-1.8	-1.8

42-Mo	Total Energy	Correlation Energy	Δ(Total Energy)	Δ(Corr. Energy)
Mo	-4047.4054	-0.1416		
Mo+2p[-1,0]+d[0]	-4047.4071	-0.1432	-1.7	-1.6
Mo+2p+1d+f[0]	-4047.4136	-0.1497	-6.5	-6.5
Mo+2p+1d+f[1]	-4047.4360	-0.1721	-28.9	-28.9
Mo+2p+1d+f[2]	-4047.4447	-0.1808	-37.6	-37.6
Mo+2p+1d+f[3]	-4047.4187	-0.1548	-11.6	-11.6
Mo+2p+1d+2f[0,1]	-4047.4366	-0.1727	-29.5	-29.5
Mo+2p+1d+2f[0,2]	-4047.4512	-0.1873	-44.1	-44.1
Mo+2p+1d+2f[0,3]	-4047.4261	-0.1622	-19.0	-19.0
**Mo+2p+1d+2f[1,2]**	**-4047.4566**	**-0.1927**	**-49.5**	**-49.5**
Mo+2p+1d+2f[1,3]	-4047.4521	-0.1882	-45.0	-45.0
Mo+2p+1d+2f[2,3]	-4047.4469	-0.1830	-39.8	-39.8
Mo+2p+1d+2f+g[2]	-4047.4576	-0.1937	-1.0	-1.0
**Mo+2p+1d+2f+g[3]**	**-4047.4596**	**-0.1957**	**-3.0**	**-3.0**
Mo+2p+1d+2f+g[4]	-4047.4593	-0.1954	-2.7	-2.7

43-Tc	Total Energy	Correlation Energy	Δ(Total Energy)	Δ(Corr. Energy)
Tc	-4284.3948	-0.1365		
Tc+2p[-1,0]+d[0]	-4284.4148	-0.1559	-20.0	-19.4
Tc+2p+1d+f[0]	-4284.4207	-0.1619	-5.9	-5.9
Tc+2p+1d+f[1]	-4284.4441	-0.1853	-29.3	-29.3
Tc+2p+1d+f[2]	-4284.4605	-0.2017	-45.8	-45.8
Tc+2p+1d+f[3]	-4284.4322	-0.1734	-17.5	-17.5
Tc+2p+1d+2f[0,1]	-4284.4451	-0.1863	-30.4	-30.4
Tc+2p+1d+2f[0,2]	-4284.4661	-0.2072	-51.3	-51.3
Tc+2p+1d+2f[0,3]	-4284.4390	-0.1802	-24.3	-24.3
**Tc+2p+1d+2f[1,2]**	**-4284.4710**	**-0.2122**	**-56.3**	**-56.3**
Tc+2p+1d+2f[1,3]	-4284.4661	-0.2073	-51.4	-51.4
Tc+2p+1d+2f[2,3]	-4284.4647	-0.2059	-50.0	-50.0
Tc+2p+1d+2f+g[2]	-4284.4739	-0.2151	-2.8	-2.8

**Tc+2p+1d+2f+g[3]**	**-4284.4758**	**-0.2170**	**-4.7**	**-4.7**
Tc+2p+1d+2f+g[4]	-4284.4744	-0.2156	-3.3	-3.3
**44-Ru**	**Total Energy**	**Correlation Energy**	**Δ(Total Energy)**	**Δ(Corr. Energy)**
Ru	-4529.4977	-0.1015		
Ru+2p[-1,0]+d[0]	-4529.5032	-0.1068	-5.6	-5.3
Ru+2p+1d+f[0]	-4529.5141	-0.1176	-10.8	-10.8
Ru+2p+1d+f[1]	-4529.5532	-0.1568	-50.0	-50.0
Ru+2p+1d+f[2]	-4529.5792	-0.1828	-76.0	-76.0
Ru+2p+1d+f[3]	-4529.5324	-0.1359	-29.1	-29.1
Ru+2p+1d+2f[0,1]	-4529.5546	-0.1582	-51.4	-51.4
Ru+2p+1d+2f[0,2]	-4529.5889	-0.1925	-85.7	-85.7
Ru+2p+1d+2f[0,3]	-4529.5445	-0.1480	-41.2	-41.2
**Ru+2p+1d+2f[1,2]**	**-4529.5976**	**-0.2011**	**-94.3**	**-94.3**
Ru+2p+1d+2f[1,3]	-4529.5902	-0.1938	-87.0	-87.0
Ru+2p+1d+2f[2,3]	-4529.5876	-0.1911	-84.3	-84.3
Ru+2p+1d+2f+g[2]	-4529.6012	-0.2048	-3.7	-3.7
**Ru+2p+1d+2f+g[3]**	**-4529.6081**	**-0.2117**	**-10.6**	**-10.6**
Ru+2p+1d+2f+g[4]	-4529.6079	-0.2115	-10.4	-10.4
**45-Rh**	**Total Energy**	**Correlation Energy**	**Δ(Total Energy)**	**Δ(Corr. Energy)**
Rh	-4782.8628	-0.1086		
Rh+2p[-1,0]+d[0]	-4782.8689	-0.1145	-6.1	-5.9
Rh+2p+1d+f[0]	-4782.8808	-0.1264	-11.8	-11.8
Rh+2p+1d+f[1]	-4782.9276	-0.1732	-58.7	-58.7
Rh+2p+1d+f[2]	-4782.9677	-0.2133	-98.8	-98.8
Rh+2p+1d+f[3]	-4782.9120	-0.1577	-43.1	-43.1
Rh+2p+1d+2f[0,1]	-4782.9293	-0.1749	-60.3	-60.3
Rh+2p+1d+2f[0,2]	-4782.9778	-0.2234	-108.9	-108.9
Rh+2p+1d+2f[0,3]	-4782.9255	-0.1711	-56.5	-56.5
**Rh+2p+1d+2f[1,2]**	**-4782.9872**	**-0.2328**	**-118.3**	**-118.3**
Rh+2p+1d+2f[1,3]	-4782.9803	-0.2259	-111.4	-111.4
Rh+2p+1d+2f[2,3]	-4782.9813	-0.2269	-112.4	-112.4
Rh+2p+1d+2f+g[3]	-4783.0007	-0.2463	-13.5	-13.5
**Rh+2p+1d+2f+g[4]**	**-4783.0013**	**-0.2470**	**-14.1**	**-14.1**
Rh+2p+1d+2f+g[5]	-4782.9916	-0.2372	-4.3	-4.3
**46-Pd**	**Total Energy**	**Correlation Energy**	**Δ(Total Energy)**	**Δ(Corr. Energy)**
Pd	-5044.6135	-0.1230		
Pd+2p[-1,0]+d[0]	-5044.6155	-0.1247	-2.0	-1.7
Pd+2p+1d+f[0]	-5044.6248	-0.1340	-9.2	-9.2
Pd+2p+1d+f[1]	-5044.6820	-0.1912	-66.5	-66.5
Pd+2p+1d+f[2]	-5044.7453	-0.2545	-129.8	-129.8
Pd+2p+1d+f[3]	-5044.6762	-0.1854	-60.6	-60.6
Pd+2p+1d+2f[0,1]	-5044.6833	-0.1925	-67.8	-67.8
Pd+2p+1d+2f[0,2]	-5044.7551	-0.2643	-139.6	-139.6
Pd+2p+1d+2f[0,3]	-5044.6883	-0.1974	-72.7	-72.7
**Pd+2p+1d+2f[1,2]**	**-5044.7671**	**-0.2763**	**-151.6**	**-151.6**
Pd+2p+1d+2f[1,3]	-5044.7585	-0.2677	-143.0	-143.0
Pd+2p+1d+2f[2,3]	-5044.7672	-0.2763	-151.6	-151.6

Pd+2p+1d+2f+g[3]	-5044.7821	-0.2913		-15.0	-15.0
**Pd+2p+1d+2f+g[4]**	**-5044.7865**	**-0.2957**		**-19.4**	**-19.4**
Pd+2p+1d+2f+g[5]	-5044.7744	-0.2835		-7.2	-7.2
Pd+2p+1d+2f+1g+s[0]	-5044.7869	-0.2960		-0.4	-0.3

**47-Ag**	**Total Energy**	**Correlation Energy**	**Davidson Corr.**	**Δ(Total Energy)**	**Δ(Corr.+Davidson)**
Ag	-5314.8593	-0.1539	-0.0090		
Ag+2p[-1,0]+d[0]	-5314.8687	-0.1608	-0.0112	-9.4	-9.1
Ag+2p+1d+f[0]	-5314.8855	-0.1751	-0.0138	-16.8	-16.8
Ag+2p+1d+f[1]	-5314.9565	-0.2399	-0.0200	-87.8	-87.8
Ag+2p+1d+f[2]	-5315.0294	-0.3110	-0.0217	-160.7	-160.7
Ag+2p+1d+f[3]	-5314.9461	-0.2347	-0.0148	-77.4	-77.4
Ag+2p+1d+2f[0,1]	-5314.9592	-0.2423	-0.0203	-90.5	-90.5
Ag+2p+1d+2f[0,2]	-5315.0444	-0.3229	-0.0249	-175.8	-175.8
Ag+2p+1d+2f[0,3]	-5314.9659	-0.2514	-0.0180	-97.3	-97.3
**Ag+2p+1d+2f[1,2]**	**-5315.0578**	**-0.3347**	**-0.0265**	**-189.1**	**-189.1**
Ag+2p+1d+2f[1,3]	-5315.0512	-0.3281	-0.0265	-182.5	-182.5
Ag+2p+1d+2f[2,3]	-5315.0558	-0.3361	-0.0232	-187.2	-187.2
Ag+2p+1d+2f+g[3]	-5315.0765	-0.3519	-0.0280	-18.7	-18.7
**Ag+2p+1d+2f+g[4]**	**-5315.0797**	**-0.3553**	**-0.0279**	**-21.9**	**-21.9**
Ag+2p+1d+2f+g[5]	-5315.0652	-0.3417	-0.0269	-7.4	-7.4

**48-Cd**	**Total Energy**	**Correlation Energy**	**Davidson Corr.**	**Δ(Total Energy)**	**Δ(Corr.+Davidson)**
Cd	-5593.5596	-0.1188	-0.0033		
Cd+2p[-1,0]+d[0]	-5593.5914	-0.1442	-0.0095	-31.8	-31.5
Cd+2p+1d+f[1]	-5593.6785	-0.2247	-0.0160	-87.1	-87.1
Cd+2p+1d+f[2]	-5593.7718	-0.3133	-0.0208	-180.4	-180.4
Cd+2p+1d+f[3]	-5593.6862	-0.2342	-0.0143	-94.8	-94.8
Cd+2p+1d+f[4]	-5593.5983	-0.1508	-0.0098	-6.9	-6.9
Cd+2p+1d+2f[1,2]	-5593.7973	-0.3355	-0.0241	-206.0	-206.0
Cd+2p+1d+2f[1,3]	-5593.7915	-0.3299	-0.0239	-200.1	-200.1
Cd+2p+1d+2f[1,4]	-5593.6932	-0.2385	-0.0170	-101.8	-101.8
**Cd+2p+1d+2f[2,3]**	**-5593.8056**	**-0.3448**	**-0.0232**	**-214.2**	**-214.2**
Cd+2p+1d+2f[2,4]	-5593.7937	-0.3331	-0.0229	-202.3	-202.3
Cd+2p+1d+2f[3,4]	-5593.6908	-0.2387	-0.0144	-99.4	-99.4
Cd+2p+1d+2f+g[3]	-5593.8274	-0.3650	-0.0247	-21.8	-21.8
**Cd+2p+1d+2f+g[4]**	**-5593.8283**	**-0.3661**	**-0.0244**	**-22.7**	**-22.7**
Cd+2p+1d+2f+g[5]	-5593.8123	-0.3511	-0.0235	-6.7	-6.7

**71-Lu**	**Total Energy**	**Correlation Energy**		**Δ(Total Energy)**	**Δ(Corr. Energy)**
Lu	-14573.7556	-0.0338			
Lu+2p[-1,0]+d[0]	-14573.7745	-0.0517		-19.0	-17.9
Lu+2p+1d+f[0]	-14573.7768	-0.0538		-2.2	-2.0
Lu+2p+1d+1f+g[1]	-14573.7770	-0.0540		-0.2	-0.2
**Lu+2p+1d+1f+g[2]**	**-14573.7775**	**-0.0545**		**-0.7**	**-0.7**
Lu+2p+1d+1f+g[3]	-14573.7772	-0.0542		-0.4	-0.4

**72-Hf**	**Total Energy**	**Correlation Energy**
Hf	-15090.1568	-0.0611

Hf+2p[-1,0]+d[0]	-15090.1765	-0.0806	-19.7	-19.5
Hf+2p+1d+f[0]	-15090.1804	-0.0844	-3.9	-3.7
Hf+2p+1d+1f+g[1]	-15090.1809	-0.0848	-0.4	-0.4
**Hf+2p+1d+1f+g[2]**	**-15090.1823**	**-0.0862**	**-1.9**	**-1.9**
Hf+2p+1d+1f+g[3]	-15090.1821	-0.0860	-1.7	-1.7
**73-Ta**	**Total Energy**	**Correlation Energy**	**Δ(Total Energy)**	**Δ(Corr. Energy)**
	sem correção			
Ta	-15618.1658	-0.1025		
Ta+2p[-1,0]+d[0]	-15618.1854	-0.1216	-19.6	-19.2
Ta+2p+1d+f[0]	-15618.1927	-0.1286	-7.4	-7.0
Ta+2p+1d+1f+g[2]	-15618.1954	-0.1313	-2.7	-2.7
**Ta+2p+1d+1f+g[3]**	**-15618.1956**	**-0.1315**	**-2.9**	**-2.9**
Ta+2p+1d+1f+g[4]	-15618.1938	-0.1297	-1.1	-1.1
**74-W**	**Total Energy**	**Correlation Energy**	**Δ(Total Energy)**	**Δ(Corr. Energy)**
W	-16157.9079	-0.1351		
W+2p[-1,0]+d[0]	-16157.9259	-0.1527	-18.0	-17.6
W+2p+1d+f[0]	-16157.9350	-0.1616	-9.1	-8.8
W+2p+1d+1f+g[2]	-16157.9384	-0.1650	-3.4	-3.4
**W+2p+1d+1f+g[3]**	**-16157.9396**	**-0.1662**	**-4.6**	**-4.6**
W+2p+1d+1f+g[4]	-16157.9369	-0.1635	-1.9	-1.9
**75-Re**	**Total Energy**	**Correlation Energy**	**Δ(Total Energy)**	**Δ(Corr. Energy)**
Re	-16709.5473	-0.2119		
Re+2p[-1,0]+d[0]	-16709.5635	-0.2278	-16.2	-15.9
Re+2p+1d+f[0]	-16709.5715	-0.2357	-8.0	-8.0
Re+2p+1d+1f+g[2]	-16709.5757	-0.2398	-4.1	-4.1
**Re+2p+1d+1f+g[3]**	**-16709.5775**	**-0.2417**	**-6.0**	**-6.0**
Re+2p+1d+1f+g[4]	-16709.5740	-0.2382	-2.5	-2.5
**76-Os**	**Total Energy**	**Correlation Energy**	**Δ(Total Energy)**	**Δ(Corr. Energy)**
Os	-17273.1250	-0.1759		
Os+2p[-1,0]+d[0]	-17273.1434	-0.1934	-18.4	-17.5
Os+2p+1d+f[0]	-17273.1705	-0.2183	-27.1	-24.9
Os+2p+1d+1f+g[2]	-17273.1770	-0.2247	-6.4	-6.4
**Os+2p+1d+1f+g[3]**	**-17273.1823**	**-0.2300**	**-11.7**	**-11.7**
Os+2p+1d+1f+g[4]	-17273.1765	-0.2242	-6.0	-6.0
**77-Ir**	**Total Energy**	**Correlation Energy**	**Δ(Total Energy)**	**Δ(Corr. Energy)**
Ir	-17848.9968	-0.1851		
Ir+2p[-1,0]+d[0]	-17849.0148	-0.2028	-18.1	-17.7

Ir+2p+1d+f[0]	-17849.0426	-0.2297		-27.8	-26.9
Ir+2p+1d+1f+g[2]	-17849.0508	-0.2379		-8.2	-8.2
**Ir+2p+1d+1f+g[3]**	**-17849.0586**	**-0.2458**		**-16.0**	**-16.0**
Ir+2p+1d+1f+g[4]	-17849.0509	-0.2380		-8.3	-8.3
**78-Pt**	**Total Energy**	**Correlation Energy**		**Δ(Total Energy)**	**Δ(Corr. Energy)**
Pt	-18437.2507	-0.2201			
Pt+2p[-1,0]+d[0]	-18437.2667	-0.2358		-16.0	-15.7
Pt+2p+1d+f[0]	-18437.2940	-0.2628		-27.3	-26.9
Pt+2p+1d+1f+g[2]	-18437.3009	-0.2697		-6.9	-6.9
**Pt+2p+1d+1f+g[3]**	**-18437.3131**	**-0.2818**		**-19.1**	**-19.1**
Pt+2p+1d+1f+g[4]	-18437.3085	-0.2773		-14.5	-14.5
**79-Au**	**Total Energy**	**Correlation Energy**	**Davidson Corr.**	**Δ(Total Energy)**	**Δ(Corr.+Davidson)**
Au	-19038.1418	-0.2831	-0.0173		
Au+2p[-1,0]+d[0]	-19038.1524	-0.2903	-0.0201	-10.6	-10.1
Au+2p+1d+f[0]	-19038.1893	-0.3207	-0.0264	-36.9	-36.6
Au+2p+1d+1f+g[2]	-19038.1982	-0.3285	-0.0274	-8.9	-8.9
**Au+2p+1d+1f+g[3]**	**-19038.2155**	**-0.3448**	**-0.0285**	**-26.2**	**-26.2**
Au+2p+1d+1f+g[4]	-19038.2087	-0.3389	-0.0276	-19.4	-19.4
**80-Hg**	**Total Energy**	**Correlation Energy**	**Davidson Corr.**	**Δ(Total Energy)**	**Δ(Corr.+Davidson)**
Hg	-19651.6669	-0.3601	-0.0411		
Hg+2p[-1,0]+d[0]	-19651.6919	-0.3257	-0.0271	-25.0	48.4
Hg+2p+1d+f[0]	-19651.7206	-0.3493	-0.0321	-28.7	-28.6
Hg+2p+1d+1f+g[2]	-19651.7312	-0.3588	-0.0332	-10.5	-10.5
**Hg+2p+1d+1f+g[3]**	**-19651.7490**	**-0.3755**	**-0.0343**	**-28.4**	**-28.4**
Hg+2p+1d+1f+g[4]	-19651.7387	-0.3661	-0.0333	-18.0	-18.0
**103 – Lr**	**Total Energy**	**Correlation Energy**		**Δ(Total Energy)**	**Δ(Corr. Energy)**
Lr	-37720.2986	-0.0308			
Lr+2p[-1,0]+d[0]	-37720.3145	-0.0451		-15.8	-14.3
Lr+2p+1d+f[0]	-37720.3171	-0.0474		-2.6	-2.3
Lr+2p+1d+1f+g[1]	-37720.3173	-0.0476		-0.3	-0.3
**Lr+2p+1d+1f+g[2]**	**-37720.3180**	**-0.0483**		**-0.9**	**-0.9**
Lr+2p+1d+1f+g[3]	-37720.3176	-0.0479		-0.5	-0.5
**104 - Rf**	**Total Energy**	**Correlation Energy**		**Δ(Total Energy)**	**Δ(Corr. Energy)**
Rf	-38707.2758	-0.0524			
Rf+2p[-1,0]+d[0]	-38707.2949	-0.0702		-19.1	-17.7
Rf+2p+1d+f[0]	-38707.3000	-0.0748		-5.0	-4.6
Rf+2p+1d+1f+g[1]	-38707.3006	-0.0754		-0.6	-0.6
**Rf+2p+1d+1f+g[2]**	**-38707.3024**	**-0.0772**		**-2.4**	**-2.4**

Rf+2p+1d+1f+g[3]	-38707.3017	-0.0765	-1.7	-1.7

**105 - Db**	**Total Energy**	**Correlation Energy**	**Δ(Total Energy)**	**Δ(Corr. Energy)**
Db	-39713.8236	-0.0881		
Db+2p[-1,0]+d[0]	-39713.8434	-0.1067	-19.8	-18.6
Db+2p+1d+f[0]	-39713.8516	-0.1142	-8.2	-7.6
Db+2p+1d+1f+g[1]	-39713.8522	-0.1149	-0.6	-0.6
**Db+2p+1d+1f+g[2]**	**-39713.8551**	**-0.1177**	**-3.5**	**-3.5**
Db+2p+1d+1f+g[3]	-39713.8549	-0.1175	-3.3	-3.3

**106 - Sg**	**Total Energy**	**Correlation Energy**	**Δ(Total Energy)**	**Δ(Corr. Energy)**
Sg	-40740.6021	-0.1173		
Sg+2p[-1,0]+d[0]	-40740.6208	-0.1348	-18.6	-17.5
Sg+2p+1d+f[0]	-40740.6319	-0.1452	-11.1	-10.4
Sg+2p+1d+1f+g[2]	-40740.6364	-0.1498	-4.5	-4.5
**Sg+2p+1d+1f+g[3]**	**-40740.6371**	**-0.1505**	**-5.3**	**-5.3**
Sg+2p+1d+1f+g[4]	-40740.6331	-0.1465	-1.3	-1.3

**107 - Bh**	Total Energy	**Correlation Energy**	**Δ(Total Energy)**	**Δ(Corr. Energy)**
Bh	-41788.3250	-0.1645		
Bh+2p[-1,0]+d[0]	-41788.3419	-0.1800	-16.9	-15.5
Bh+2p+1d+f[0]	-41788.3555	-0.1929	-13.6	-12.9
Bh+2p+1d+1f+g[2]	-41788.3616	-0.1989	-6.1	-6.1
**Bh+2p+1d+1f+g[3]**	**-41788.3629**	**-0.2003**	**-7.5**	**-7.5**
Bh+2p+1d+1f+g[4]	-41788.3573	-0.1947	-1.8	-1.8

**108 - Hs**	**Total Energy**	**Correlation Energy**	**Δ(Total Energy)**	**Δ(Corr. Energy)**
Hs	-42856.7714	-0.1729		
Hs+2p[-1,0]+d[0]	-42856.7862	-0.1864	-14.7	-13.5
Hs+2p+1d+f[0]	-42856.8008	-0.2006	-14.6	-14.2
Hs+2p+1d+1f+g[2]	-42856.8100	-0.2098	-9.2	-9.2
**Hs+2p+1d+1f+g[3]**	**-42856.8138**	**-0.2137**	**-13.0**	**-13.0**
Hs+2p+1d+1f+g[4]	-42856.8043	-0.2042	-3.5	-3.5

**109 - Mt**	**Total Energy**	**Correlation Energy**	**Δ(Total Energy)**	**Δ(Corr. Energy)**
Mt	-43947.2687	-0.1687		
Mt+2p[-1,0]+d[0]	-43947.2835	-0.1823	-14.7	-13.6
Mt+2p+1d+f[0]	-43947.3220	-0.2181	-38.5	-35.9
Mt+2p+1d+1f+g[2]	-43947.3327	-0.2289	-10.7	-10.7
**Mt+2p+1d+1f+g[3]**	**-43947.3398**	**-0.2360**	**-17.8**	**-17.8**
Mt+2p+1d+1f+g[4]	-43947.3272	-0.2233	-5.2	-5.2

**110 - Ds**	**Total Energy**	**Correlation Energy**	**Δ(Total Energy)**	**Δ(Corr. Energy)**
Ds	-45057.8606	-0.2023		
Ds+2p[-1,0]+d[0]	-45057.8746	-0.2149	-14.0	-12.7

Ds+2p+1d+f[0]	-45057.9142	-0.2528		-39.7	-37.9
Ds+2p+1d+1f+g[2]	-45057.9265	-0.2651		-12.3	-12.3
**Ds+2p+1d+1f+g[3]**	**-45057.9363**	**-0.2749**		**-22.1**	**-22.1**
Ds+2p+1d+1f+g[4]	-45057.9205	-0.2591		-6.3	-6.3
**111 - Rg**	**Total Energy**	**Correlation Energy**	**Davidson Corr.**	**Δ(Total Energy)**	**Δ(Corr.+Davidson)**
Rg	-46193.3314	-0.2404	-0.0144		
Rg+2p[-1,0]+d[0]	-46193.3488	-0.2520	-0.0188	-17.4	-16.0
Rg+2p+1d+f[0]	-46193.3938	-0.2893	-0.0256	-45.0	-44.1
Rg+2p+1d+1f+g[2]	-46193.4076	-0.3016	-0.0270	-13.8	-13.8
**Rg+2p+1d+1f+g[3]**	**-46193.4235**	**-0.3164**	**-0.0282**	**-29.8**	**-29.8**
Rg+2p+1d+1f+g[4]	-46193.4036	-0.2983	-0.0263	-9.8	-9.8
**112 - Cn**	**Total Energy**	**Correlation Energy**	**Davidson Corr.**	**Δ(Total Energy)**	**Δ(Corr.+Davidson)**
Cn	-47352.4426	-0.2841	-0.0177		
Cn+2p[-1,0]+d[0]	-47352.4595	-0.2953	-0.0219	-16.9	-15.4
Cn+2p+1d+f[0]	-47352.5024	-0.3305	-0.0290	-42.9	-42.3
Cn+2p+1d+1f+g[2]	-47352.5141	-0.3411	-0.0302	-11.7	-11.7
**Cn+2p+1d+1f+g[3]**	**-47352.5345**	**-0.3597**	**-0.0319**	**-32.1**	**-32.1**
Cn+2p+1d+1f+g[4]	-47352.5141	-0.3413	-0.0300	-11.7	-11.7
